# Perceived Stress and Life Satisfaction Among Chinese Clinical Nursing Teachers: A Moderated Mediation Model of Burnout and Emotion Regulation

**DOI:** 10.3389/fpsyt.2021.548339

**Published:** 2021-07-08

**Authors:** Xia Xu, Ling Chen, Yuan Yuan, Ming Xu, Xiaocui Tian, Fang Lu, Zonghua Wang

**Affiliations:** ^1^Department of Nursing, Daping Hospital, Chongqing, China; ^2^Department of Emergency, The 958th Hospital of PLA, Chongqing, China; ^3^Department of Clinical Nursing Research and Teaching, Southwest Hospital, Chongqing, China; ^4^Department of Health Management, Daping Hospital, Chongqing, China; ^5^Department of Neurology, Daping Hospital, Chongqing, China; ^6^Department of Nursing Management, School of Nursing, Army Medical University, Chongqing, China; ^7^Department of Field Nursing, School of Nursing, Army Medical University, Chongqing, China; ^8^Department of Clinical Nursing, School of Nursing, Army Medical University, Chongqing, China

**Keywords:** perceived stress, burnout syndrome, emotion regulation, life satisfaction, clinical nursing teachers

## Abstract

Our previous study indicated that clinical teaching nurses in China suffered high levels of perceived stress and burnout, mainly because they were taking double responsibilities of nursing and teaching at the same time. The study aimed to investigate the underlying mechanisms of how and when perceived stress increased the risk of burnout and decreased life satisfaction among clinical teaching nurses. Questionnaires about perceived stress, burnout, emotion regulation, and life satisfaction were self-administered to 1,372 teaching nurses from eight tertiary military hospitals in China. Correlation and hierarchical multiple regressions were employed for data analysis. The results revealed that perceived stress had direct and indirect impacts on life satisfaction, with the principal element of burnout—emotional exhaustion—acting as a mediator. Moreover, the association between perceived stress and emotional exhaustion was moderated by emotion suppression—a key emotion regulation strategy. The negative impact of perceived stress on burnout was stronger among teaching nurses with high emotion suppression than among those with low emotion suppression. The present study contributed to a deeper understanding of the relationship between perceived stress and life satisfaction and also suggested further research into emotion regulation interventions to alleviate or eliminate the impact of perceived stress on burnout and eventually improve the life satisfaction for Chinese clinical nursing teachers.

## Introduction

Clinical teaching is a requisite part of nurse education. Through clinical training, nursing students are expected to successfully transit from students in classrooms into nurses in clinical settings and to develop abilities and skills essential for providing the best possible care to their patients. In other words, good quality of clinical education guarantees the quality of the nursing care and eventually impacts patients' health outcomes. Undoubtedly, clinical nursing teachers play a core role in clinical teaching. Their clinical expertise and teaching strategies greatly impact the quality and outcomes of clinical education. Therefore, most current studies have been focused on role transitions (1, 2) and preparation of clinical nursing teachers to promote competence and confidence in clinical teaching (3).

Nursing is considered a strenuous job with complicated demands, which leads to occupational stress. In a study of investigating stress and associated risk factors among 2,895 Iran nurses, 78.4% of the respondents reported a high level of occupational stress (3). The risk factors for high stress included increased work hours and working in emergency, intensive care wards, and teaching hospitals. Clinical nurses working in teaching hospitals were regarded as particularly vulnerable compared to those working in non-teaching hospitals; they reported greater job stress and dissatisfaction and higher intention to leave (3). Clinical nursing teachers frequently complained that clinical expert knowledge and skills could not always translate into clinical teaching expertise (4). Their stresses got doubled during the preparation of being “double-certified” and expertise in two different professions: nursing and education (4). Clinical nurses have already struggled with high patient care demands, lack of resources, long work hours, heavy workloads, and even workplace violence (5, 6); they have also been overwhelmed by responsibilities associated with clinical teaching. Other sources of stress for nurse educators included insufficient time for teaching preparation (7), role conflicts with peers and supervisors, strict appraisal standards for teaching abilities, pressure of conducting scientific researches, worries about accidents at work, and so on.

Work-related stress has been implicated as a major factor for mental health difficulties such as compassion fatigue, burnout syndromes, anxiety, and reduced job satisfaction, which negatively affected life satisfaction (8) and quality of life (QOL) (9–11). These stress-related problems reduced work productivity, increased incidence of practice errors, and were unfavorably associated with quality of health service (12). Moreover, occupational stress has been recognized as a risk factor for psychiatric sickness absence (13), intention to leave (14), and eventually nurse shortage (15). On the other hand, stress and its poor outcomes among clinical nursing teachers could result in negative impacts on role preparedness and confidence in clinical teaching and eventually reduced the effectiveness of clinical teaching and quality of education outcomes (16).

### Burnout Symptom as a Mediator

Occupational stress can not only directly damage life satisfaction but also indirectly reduce life satisfaction through mediating variables, such as burnout. Burnout was one of the most common psychological disorders among healthcare professionals, in particular among nursing workers. Described as a state of emotional fatigue and exhaustion, burnout was caused by repeated and prolonged exposure to stressful working environments and situations. A long-term status of burnout resulted in a decrease or loss of motivation for work that can elicit exhaustion and a sense of failure (17, 18). According to Maslach's burnout model, three core characteristics were illustrated: depersonalization (DP), prolonged emotional exhaustion (EE), and reduced personal accomplishment (PA) at work (19, 20). There was a high prevalence of burnout among healthcare workers, in particular among nurses who cared for critically ill patients. Professor Li and Dr. Lu investigated on intensive care unit (ICU) nurses and found out that 84% of the participants have at least one aspect of burnout symptoms: 23% were positive for EE, 27% were positive for DP, and 77.8% were positive for lack of PA (19, 20).

Higher perceived stress was closely connected to higher levels of burnout. A study among 366 female nurses revealed that 85.5% of the participants experienced psychological distress and that burnout was positively associated with psychological distress (21). A high positive correlation between job stress and burnout has been found among 79 head nurses (*r* = 0.426, *p* < 0.01) and 145 senior nurses (*r* = 0.554, *p* < 0.01) (22). Similar results were found in nurse teachers. Our previous study has investigated on perceived stress and burnout among 835 nurse teachers in military hospitals. The results have revealed high rates in three aspects of burnout: loss of passion for work (18.8%), DP (12.5%), and reduced PA (28.1%). These aspects of burnout have a significant positive correlation with perceived stress, with correlation coefficients ranging from 0.28 to 0.48. Moreover, perceived stress has been demonstrated as a predictor of burnout through a multiple stepwise regression analysis (22).

Emerging evidence has suggested that associations may exist between occupational burnout and decreased life satisfaction (10). As an important component of subjective wellbeing (SWB), life satisfaction referred to a comprehensive evaluation of satisfaction and happiness about people's own overall life (23). Compared to other primary healthcare professionals, nurses demonstrated higher perceived stress, lower positive affect, and lower levels of mindfulness (24). A national study across China has been conducted to examine the association of job burnout with life satisfaction among 7,289 employees. One-third of the participants reported decreased life satisfaction. The logistic regression analysis showed that EE, DP, and reduced sense of PA were risk factors for low life satisfaction (*p* < 0.001) (25). In another study, registered nurses working in critical care units reported moderate to high levels of burnout and low levels of job satisfaction; and burnout acted as a predictor of the decreased job satisfaction (26).

### Emotion Regulation as a Moderator

Emotion regulation (ER) was a process to evaluate and control expression of emotions. It reflected an individual's psychosocial adaptation, emotional flexibility, and coping capability to a stressful situation. Gross's process model of ER pointed out that two of the most widely used strategies were cognitive reappraisal (CR) and expressive suppression (ES). CR concentrates on the emotion generative process, referring to a reinterpretation to the situation so as to alter its emotional response. ES addresses the response tendency, referring to individuals' tendency of inhibiting or controlling emotional expressions and behavioral reactions. According to the transactional model of stress (27, 28), individual's perceived stress depended on the process of cognitive appraisal and ER, rather than the event itself. This process determined emotional responses and eventually affected life satisfaction and SWB. Therefore, the relationship of stress, burnout, and life satisfaction was assumed to be moderated by ER strategies.

Emotion regulation can modify the effects of stressful events. Lack of ER capacity was associated with mental health disorders and may result in decreased life satisfaction and QOL among healthcare workers. Individuals with negative ER skills were prone to experience negative emotions such as anxiety, EE, and insufficient feeling of job accomplishment. What remained unclear was the role of ER in the association between perceived stress and burnout among clinical nursing teachers.

### Aim of the Present Study

Although it is widely acknowledged that nurses have been subjected to stress, burnout, and decreased life satisfaction (29, 30), few literature have investigated the perceived stress and burnout among teaching nurses and the mechanisms of how perceived stress influences burnout and life satisfaction. Such an investigation is imperative for clinical nurse teachers in order to develop strategies to manage stress, prevent burnout syndromes, improve mental health outcomes, and promote overall QOL. Therefore, this study aimed to (1) explore the relationship of perceived stress with burnout and life satisfaction among Chinese clinical nursing teachers and (2) examine a moderated mediation model of this relationship. Firstly, we investigated the mediating effect of burnout syndromes on the relationship between perceived stress and life satisfaction. Secondly, we explored the moderating effect of ER on the relationship between perceived stress and life satisfaction via the mediation of burnout syndromes.

## Method

### Participants

This cross-sectional survey was conducted between June 2018 and May 2019. A convenience sampling procedure was used to recruit clinical nurse educators from eight military tertiary hospitals. These hospitals were teaching hospitals affiliated to the only three military medical universities across China, which were an army medical university, navy medical university, and air force medical university. Our researchers contacted nurse managers to help us release adverts to clinical nurses and invited them to participate if they met the following inclusion criteria: (1) working as a nurse in clinics and hospitals; (2) getting trained and certified on teaching; (3) undertaking clinical teaching in at least one nursing course; and (4) giving consent to participate. Participants were excluded if they (1) failed to reach a total teaching hour of 160 annually and (2) were not involved in clinical teaching for more than 1 year.

### Measures

#### Perceived Stress

The Chinese Perceived Stress Scale (CPSS) revised by Yang and Huang (31) was adopted to evaluate perceived stress in one's life over the past month. This scale included 14 items in two dimensions: sense of control (seven items) and sense of tension (seven items), rating on a 5-point Likert scale (0 = “not at all”; 6 = “always”). The total score ranged from 0 to 56, with higher scores indicating a higher level of perceived stress. The internal consistency coefficient was 0.82, 0.86, and 0.77 for the total scale and the subscales of “sense of control” and the “sense of tension,” respectively (32). A CPSS ≥ 26 could be judged as having health risk pressure (33). In our subjects, the internal consistency value of the α coefficient was 0.67.

#### Burnout

The Maslach Burnout Inventory-Human Service Survey (MBI-HSS) was the most commonly used scale to evaluate burnout syndromes among healthcare professionals. The Chinese version of MBI was translated and validated by Professor Peng Mei-ci from the Hong Kong Polytechnic University and was published in the book of Shang et al. (34). The 22-item MBI-HSS scale included three subscales: a nine-item subscale of EE, a five-item subscale of DP, and an eight-item subscale of PA. Each item evaluated the degree of burnout experience and feeling on a 7-point Likert scale (0 = “never”; 6 = “every day”). The total scores on the EE and DP subscales were positively related to burnout, while the scores on PA were inversely related to burnout. According to Maslach et al., this study defined “low burnout” by scores on EE of ≤ 16, on DP of ≤ 6, and on PA of ≥39; “moderate burnout” by scores on EE ranging from 17 to 26, on DP ranging from 7 to 12, and on PA ranging from 38 to 32; and “high burnout” by scores on EE of ≥27, on DP of ≥13, on and PA of ≤ 31. In our participants, Cronbach's α score of each domain ranged from 0.70 to 0.85, with Cronbach's α coefficient for the overall scale of 0.74.

#### Emotion Regulation

The Emotion Regulation Questionnaire (ERQ) developed by Gross (35) was used to evaluate participants' ER strategies. A Chinese version translated and validated by Wang et al. (36) was adopted in this study. This questionnaire was composed of a six-item CR subscale and a four-item “expression suppression” (ES) subscale. A 7-point Likert rating method was adopted, with 1 to 7 representing “strongly disagree” to “strongly agree.” The alpha reliability was 0.79 for “cognitive reappraisal” and 0.73 for ES, respectively. Test–retest reliability across 3 months was 0.69 for both subscales. Cronbach's α value was 0.75 in our sample.

#### Life Satisfaction

The Satisfaction with Life Scale (SWLS) developed by Diener et al. (37, 38) offered a global measure of satisfaction with life and SWB. It comprised five items answered with a 7-point Likert scale ranging from “1 = totally disagree” to “7 = totally agree.” Higher scores represented greater perceived life satisfaction. The Chinese version of SWLS indicated good validity and high internal consistency reliability (Cronbach's alpha = 0.84) (39). Total scores can be categorized as follows: very high scores/highly satisfied (30–35 points), high scores (25–29 points), average scores (20–24 points), slightly below average in LS (15–19 points), dissatisfied (10–14 points), and extremely dissatisfied (5–9 points) (40). Cronbach's α value was 0.89 in this study.

### Procedure

The participants filled out a self-administered questionnaire in an anonymous way after reading written instructions and giving informed consent through an online platform. A total of 1,395 questionnaires were collected, in which 1,372 were valid for further analysis. This study was conducted in accordance with the ethical principles of the Declaration of Helsinki. Ethical approval was obtained from the human ethics committee for Scientific Research at the first researcher's university.

### Data Analysis

The software SPSS (version 21.0) and the Hayes SPSS macro program PROCESS (version 3.2) were employed to organize and analyze the data, including the nature of the moderated indirect effects. Descriptive statistics and bivariate correlation analysis were first conducted. All regression coefficients were tested by the bias-corrected percentile bootstrap method. This method produced 95% confidence interval (CI) for these effects from 5,000 bootstrap samples; 95% CIs that do not include 0 indicated a significant effect. In the current study, we selected the mediation model (Model 4) to analyze mediation effects.

## Results

### Sample Characteristics

The participants consisted of 1,348 females and 24 males. Regarding professional titles, 63.8% (876/1,372) held a primary level, 31.7% (435/1372) held a mediate level, and only 3.9% (53/1,372) held an advanced level. Among all of the participants, 12.8% (176/1372) have been working in the hospital for less than 5 years; 44.5% (611/1,372) for 5–10 years; 22.6% (310/1,372) for 11 to 15 years; 13.2% (181/1,372) for 16–20 years; and 6.9% (94/1,372) for more than 20 years. The average length of clinical teaching experience was 3.06 ± 1.38 years.

### Descriptive Analysis

There is a prevalence of burnout syndrome according to Maslach's cutoff of burnout and its dimensions illustrated in the Method section. Almost 80% (1,091/1,372) of participants showed a moderate degree of burnout, and 5% (65/1,372) indicated a high degree of burnout. Regarding to each dimension of burnout, 52% (714/1,372) of the clinical nursing teachers were positive for moderate to high levels of EE, 28.9% (396/1,372) were positive for moderate to high levels of DP, and 74.5% (1,036/1,372) were positive for moderate to high levels of insufficient PA. The mean scores of perceived stress were 39.32, higher than the cutoff score of 26, indicating significant health risk pressure; the mean scores of SWB was 21.03, a little higher than the midpoint of 20 (see Table 1).

**Table 1:** Descriptive statistics and bivariate correlations of the major study variables (*n* = 1,372).

**Variables**	***M* ±*SD***	**1**	**2**	**3**	**4**	**5**	**6**	**7**
1. Perceived stress (PSS)	39.32 ± 6.52	1						
2. Emotional exhaustion (MBI_EE)	18.50 ± 10.48	0.483^**^	1					
3. Depersonalization (MBI_DP)	5.15 ± 4.82	0.307^**^	0.552^**^	1				
4. Personal achievement (MBI_PA)	31.12 ± 9.37	−0.395^**^	−0.170^**^	−0.234^**^	1			
5. Cognitive reappraisal (ERQ_CR)	30.22 ± 6.18	−0.322^**^	−0.153^**^	−0.206^**^	0.378^**^	1		
6. Expression suppression (ERQ_ES)	14.35 ± 4.53	0.118^**^	0.114^**^	0.117^**^	0.001	0.110^**^	1	
7. Life satisfaction (SWLS)	21.03 ± 6.39	−0.498^**^	−0.339^**^	−0.231^**^	0.330^**^	0.322^**^	0.010	1

*PSS, Perceived Stress Scale; MBI, Maslach Burnout Inventory–Human Service; ERQ, Emotion Regulation Questionnaire; SWLS, Satisfaction with Life Scale*.

** *p < 0.01*.

### Correlation Analysis

Table 1 also presented the results of bivariate correlations between different main variables. The perceived stress was significantly positively correlated with EE (*r* = 0.483), DP (*r* = 0.307), and the ER strategy of ES (*r* = 0.118) and negatively associated with PA (*r* = −0.395), the ER strategy of CR (*r* = −0.322), and SWB (*r* = −0.498) (all *p* < 0.01). SWB was significantly positively associated with PA (*r* = 0.330) and CR (*r* = 0.322) and negatively related to EE (*r* = −0.339) and DP (*r* = −0.231; all *p* < 0.01).

### Mediation Effect Analysis

In the SPSS macro PROCESS software, the analysis of mediating effect was conducted by Model 4 to investigate EE on the relationship between perceived stress and SWB. Model 1 in Table 2 indicated that the perceived stress was a significantly negative predictor on SWB (*B* = 0.487, *t* = −21.249, and *p* < 0.001). It was suggested in Model 2 that perceived stress was a significantly positive predictor of EE (*B* = 0.776, *t* = 20.396, and *p* < 0.001), while EE had a significantly negatively predicted effect on SWB (*B* = −0.078, *t* = −4.843, and *p* < 0.001). Moreover, as shown in Model 3, the direct effect of perceived stress on SWB was still significant (*B* = −0.427, *t* = −16.425, and *p* < 0.001) when mediating variables were added. In addition, the upper and lower bounds of the bootstrap 95% CI for the direct effect of perceived stress on SWB and the mediating effect of EE did not include 0, indicating that the mediating effect was significant. The mediation effect accounted for 26.1% of the total effect, which confirmed that EE played a partial mediating role in the relationship between perceived stress and SWB.

**Table 2:** Mediation analysis (*n* = 1,372).

**Variables**	**Model 1 (SWLS)**				**Model 2 (MBI_EE)**				**Model 3 (SWLS)**			
	**β**	***t***	**Bootstrap 95%CI**		**β**	***t***	**Bootstrap 95%CI**		**β**	***t***	**Bootstrap 95%CI**	
			**LLCI**	**ULCI**			**LLCI**	**ULCI**			**LLCI**	**ULCI**
PSS	−0.487	−21.249^***^	−0.532	−0.442	0.776	20.396^***^	0.701	0.850	−0.427	−16.425^***^	−0.478	−0.376
MBI_EE									−0.078	−4.843^***^	−0.110	−0.047
*R*^2^	0.248				0.233				0.261			
F	451.502^**^				415.995^***^				241.176^***^			

*PSS, Perceived Stress Scale; EE, Emotional Exhaustion; SWLS, Satisfaction with Life Scale*.

** *p < 0.01*,

*** *p < 0.001*.

### Moderated Mediation Effect Analysis

As shown in Table 3, after the demographic variables of age and marital status were controlled for, the entry of perceived stress in step 2 indicated that perceived stress was positively associated with EE. After the entry of ES in step 3, the result revealed a unique variance in EE while controlling for perceived stress, age, and marital status.

**Table 3:** Hierarchical multiple regression analyses of perceived stress and expression suppression on emotional exhaustion (*n* = 1,372).

**Steps and independent variables**	**Emotional exhaustion**		
	**β**	**Total R^**2**^**	**Δ*R*^**2**^**
**Step 1**			
Age	−0.74		
Marital status	−0.43	0.010	0.008
**Step 2**			
Perceived stress	0.478	0.234	0.233
**Step 3**			
Expression suppression	0.090	0.242	0.240
**Step 4**			
Perceived stress × expression suppression	0.062	0.246	0.243

**Figure 1:**
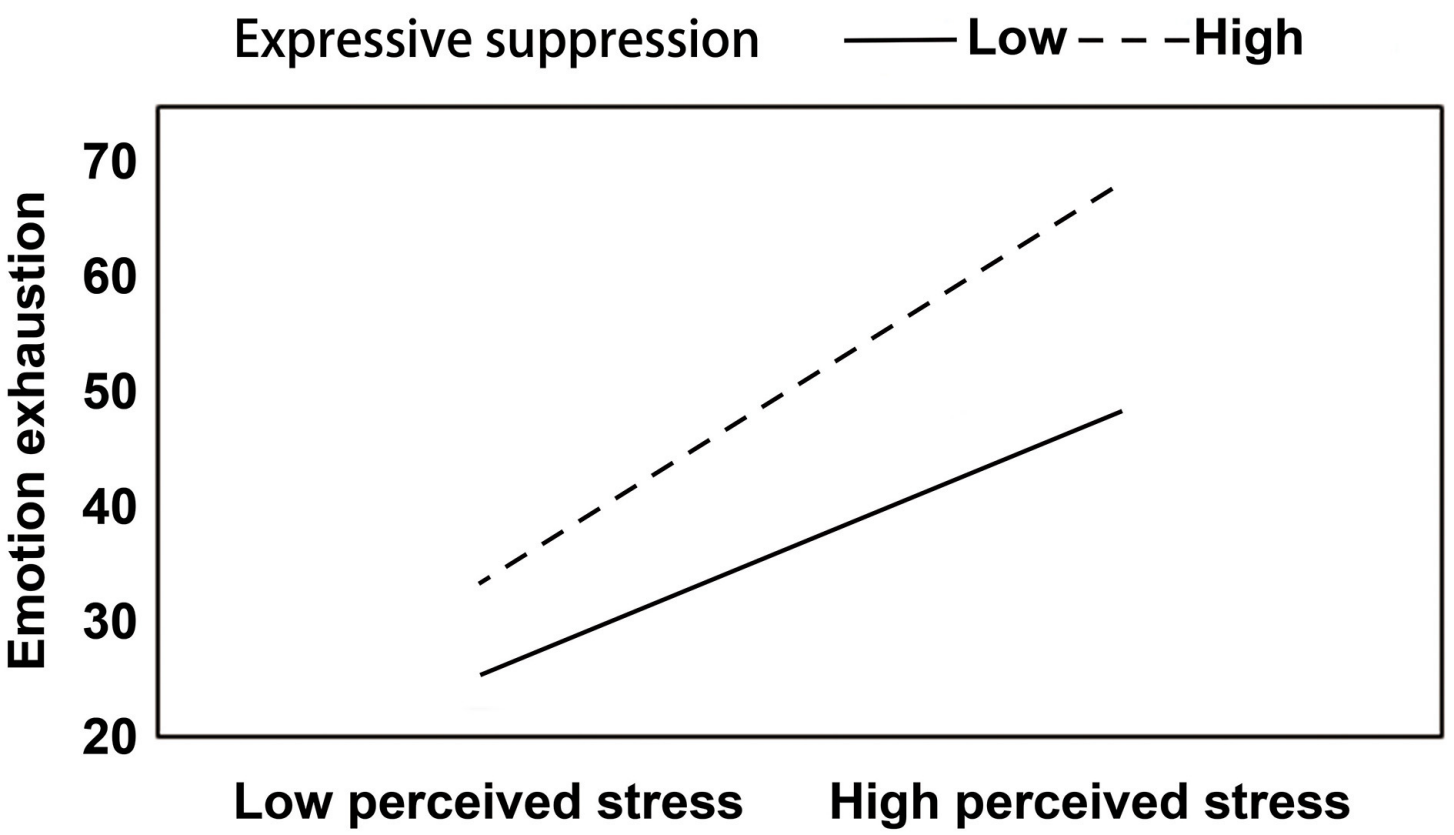
The moderating effect of expression suppression on the relation between perceived stress and emotional exhaustion.

The entry of the interaction term perceived stress × ES in step 4 results in a significant result related to EE. This indicated that ES strengthened the effect of perceived stress on EE. A simple slope analysis showed that perceived stress predicted EE at both low (*B* = 0.477, *t* = 5.131, and *p* < 0.001) and high (*B* = 0.732, *t* = 8.187, and *p* < 0.001) levels of ES, but the association between perceived stress and EE was stronger when the ES level was high (see Figure 1).

## Discussion

**Figure 2:**
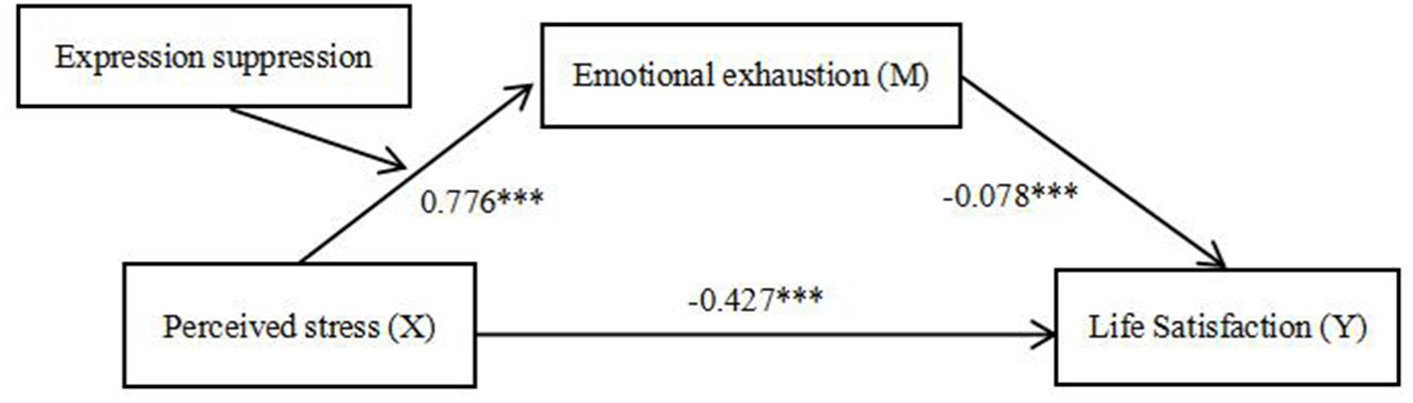
The finalized moderated mediation model (*n* = 1,372). ***p < 0.001.

During the past decades, an increasing number of research has been published in terms of burnout and SWB among clinical nurses. The mental health and overall QOL of clinical nursing teachers, as a special group of nurses, required increasing concerns. This study investigated the relationship of perceived stress with burnout and life satisfaction. Consistent with previous research, our research added evidence to confirm direct and indirect impacts of perceived stress on life satisfaction among Chinese nursing teachers (9). Moreover, to our best knowledge, this study was the first to propose a moderated mediation model to analyze the mechanism underlying the association between perceived stress, job burnout, ER, and life satisfaction. According to previous empirical studies and theories, we proposed a model that incorporated the mediating variable of burnout and the moderating variable of ER ability in the relationship between perceived stress and life satisfaction (Figure 2).

In particular, our findings indicated that the mediating role of burnout on the indirect relationship between perceived stress and life satisfaction was significant in the dimension of EE. The perceived stress was a significantly positive predictor of EE. EE, as the most core element in the Maslach burnout model, was regarded as feelings of being emotionally drained and depleted of one's emotional resources (41). Just as Maslach et al. (42) stated, “when people describe themselves as experiencing burnout, they are most often referring to the experience of emotional exhaustion”. In addition, it has been shown that EE could result in other characteristics of burnout: DP and cynicism (42). The significance of EE could be predicted and explained according to Hans Selye's stress model of General Adaptation Syndrome (GAS). Hans Selye's stress model was a three-stage response that occurred when people encountered stress: alarm, resistance, and exhaustion. EE was an inevitable outcome when the stress response proceeded to the end stage. EE occurred if long-term stress continued beyond coping capacities and one's adaptation, which further increased the likelihood of suffering from a decreased level of life satisfaction. Khoo et al. illustrated in their multicenter survey that stress in the workplace was the most significant factor for EE through multivariate analysis. About three-quarters of 38 sources of job stress were significantly associated with EE; and the most common source of stress was dealing with difficult parents (80.2%) (43).

Another finding worthy of note was that the perceived stress not only had a direct effect on EE but also had an indirect influence on EE, via mediation by the ER strategy of ES. The effect of perceived stress on EE could be strengthened by ES. For nursing teachers with high-level ES, the impact of perceived stress on EE was stronger than those with low-level ES. In other words, the more likely that clinical nursing teachers adopted an improper strategy of ER such as ES, the more they were vulnerable to suffer from EE. ES was a response-focused strategy, referring to individuals' efforts to suppress the expression or experience of emotion, especially in an attempt to control the behavioral component of the emotional response. For example, individuals with a high level of ES tended to “try to behave in such a way that a person watching you would not know you were feeling anything” (44). The association of ES with negative indicators of well-being was evident in previous studies. For individuals with cancer, those who tended to use more ES had reported lower QOL across all aspects (45).

The following reasons may explain the significance of ES among nursing teachers. The first reason might be the stressful situations that they have confronted. They were fully occupied with various sources of demands and problems, from both patients and intern students so that they have limited time and opportunities to consider and express their own feelings, emotions, and thoughts. Secondly, the implicit communication style in Chinese culture may account for the emotion expression among Chinese clinical nursing teachers. Influenced by Confucian philosophy, the Chinese are socialized to control the expression of their emotions and repress overt feelings. Only in this way could nursing teachers maintain professional authority among students. The third reason was the preference and role requirements of adopting emotional labor strategies among most nurses (46). Based on Hochschild's work (47), emotional labor strategies mainly comprised surface acting and deep acting. Surface acting stands for suppression of one's felt emotions, while deep acting represents an attempt to change one's felt emotions to meet the role demands (48). Suppression and control of emotion expression were thought to be a desired emotional state for nurses. They worried that too much emotional engagement with patients and students may disrupt medical equity and lead to a preference during decision making and practice. However, stress came up when nurses felt a mismatch between their actual feelings and displayed emotions during their interactions with patients and students, which was confirmed to result in burnout and negatively affected life satisfaction and wellbeing (48).

The study has significant theoretical and practical implications. Firstly, the results may help explain the underlying mechanisms of relationships among perceived stress, burnout, ER, and life satisfaction. Secondly, given the mediation role of ES, this study may inform initiatives to carry out ER training (ERT) to strengthen stress management. Generally, ERT incorporates techniques including but not limited to mindfulness (49), cognitive-behavioral therapy (50), and emotion-focused mediation (51) in order to improve ER skills to deal with stressful situations. ERT is expected to reduce or eliminate burnout syndromes and improve life satisfaction among workers with highly occupational stress such as clinical teaching nurses. Previous studies reveal that mental health (52) and professional QOL (53) can be increased through ERT among intensive and critical care nurses. Therefore, further research should be designed and conducted to examine the effectiveness of ERT that aims to alleviate or eliminate the impact of perceived stress on burnout and eventually improve life satisfaction for Chinese clinical nursing teachers.

In addition to its strengths, several limitations should be pointed out. Firstly, our sample was nurses all recruited from military hospitals. Considering that they were also taking responsibilities of military missions and disaster rescues, this group of nurses was regarded as having a higher level of perceived stress and burnout compared to nurses working in civilian hospitals. Besides, it was hard to determine that their stress was only from duties as clinical teachers and not from their duties as military nurses. Further research was required to explore the exact factors contributing to their perceived stress, in order to develop targeted interventions of stress management in this group of nurses. Secondly, this study failed to demonstrate the cause and effect relationships among the variables as a cross-sectional design was employed. For this reason, future studies might wish to apply a longitudinal or an experimental research design. Thirdly, it should be noted that the bias might occur when we used a cross-sectional study design to explore the mediational processes, although most of the previous studies on mediation analysis did not consider the role of time. However, as early as the year of 1981, Dr. Judd and Kenny have declared the potential importance of studying meditation from a longitudinal design. They emphasized that failing to include prior assessments of the mediator and the outcome could lead to bias. In this respect, a longitudinal study was required in the future to give a more accurate examination of the mediation model (54).

## Conclusion

This study investigated the mechanisms underlying associations between perceived stresses, job burnout, and life satisfaction among Chinese clinical teaching nurses using the moderated mediation model. The results indicated the direct and indirect relationships of perceived stress with burnout symptoms and life satisfaction. In particular, the findings demonstrated the mediator role of the principal element of burnout—EE—in the association between stress and life satisfaction. And ES partially moderated the strength of the relationship between perceived stress and job burnout. As a result, ERT was implied as a useful intervention to alleviate or eliminate the impact of perceived stress on burnout and eventually improve the life satisfaction for Chinese clinical nursing teachers.

## Data Availability Statement

The raw data supporting the conclusions of this article will be made available by the authors, without undue reservation.

## Ethics Statement

Ethical approval was obtained from the Ethics Committee of Daping Hospital affiliated to Army Medical University (number: 181).

## Author Contributions

XX, ZW, and FL: study design. XX, LC, YY, MX, and XT: data collection. ZW, LC, and FL: data analysis. ZW and FL: manuscript writing. All authors contributed to the article and approved the submitted version.

## Conflict of Interest

The authors declare that the research was conducted in the absence of any commercial or financial relationships that could be construed as a potential conflict of interest.
